# Impact of Faculty Incentivization on Resident Evaluations

**DOI:** 10.5811/westjem.59267

**Published:** 2023-07-12

**Authors:** Viral Patel, Alexandra Nordberg, Richard Church, Jennifer L. Carey

**Affiliations:** University of Massachusetts Chan Medical School, Department of Emergency Medicine, Worcester, Massachusetts

## Abstract

**Introduction:**

In the Program Requirements for Graduate Medical Education in Emergency Medicine, the Accreditation Council for Graduate Medical Education requires frequent and routine feedback. It is a common challenge for program leadership to obtain adequate and effective summative evaluations.

**Methods:**

This is a retrospective, case-crossover, interventional study conducted in an academic medical center. This study occurred over a two-year period, with an intervention between years one and two. Throughout year two of the study, faculty incentive compensation was linked to completion of end-of-shift evaluations. We compared pre- an post-implementation data using paired sample *t*-tests with the significance level *P* < .05 applied.

**Results:**

After implementation of the incentive metric there was an increase in the number of total evaluations by 42% (*P* = .001). The mean number of evaluations submitted by each faculty per shift increased from 0.45 to 0.86 (SD 0.56, *P* < .001). Overall, 32 of the 38 faculty members (84.2%) had an increase in the number of evaluations submitted per shift during the intervention period with an average increase of 0.5 evaluations per shift (range 0.01–1.54).

**Conclusion:**

Incentivizing faculty to submit resident evaluations through use of bonus compensation increased the number of evaluations at our institution. This information may be applied by other programs to increase resident evaluations.

## INTRODUCTION

The Accreditation Council for Graduate Medical Education (ACGME) requires emergency medicine (EM) residency programs to obtain frequent feedback for resident physicians that is both summative and formative.^1^ To accomplish this, many programs employ an evaluation form that is to be completed by faculty at the conclusion of each resident shift.^2^,^3^ This formative feedback is compiled by clinical competency committees to create summative feedback for residents, comparing each resident against milestones and their peers. This assists the program director in making evaluative decisions regarding the performance and abilities of trainees.

Consistently obtaining end-of-shift feedback from faculty has been a targeted area of improvement within medical training.^2^,^4^–^7^ Different strategies have been executed, with digital collection being shown to improve faculty response rates compared to paper systems.^8^,^9^ Yet even with such implementation there is still a lack of sufficient feedback collection to construct a global, summative evaluation of residents in all milestones.^2^,^10^ Strategies have been implemented to improve faculty engagement and participation in educational campaigns at the undergraduate medical education and graduate medical education (GME) levels.^2^,^4^–^7^ There is evidence that financially based incentivization has led to improved faculty participation in learner evaluation, notably on a monthly or end-of-rotation basis.^7^ Therefore, we hypothesized that employing such an incentivization process would increase the overall quantity of resident end-of-shift evaluations completed by faculty.

Our study addresses the ACGME requirement for increased frequency of evaluation, effectively on a near-daily basis, and shows that financial strategies can motivate faculty to engage in this level of participation. Our study appears to be the first of its kind in directing attention to the requirements necessary for the provision of extensive, timely feedback to our learners.

## METHODS

This was a retrospective, case-crossover interventional study conducted at an academic EM residency training site. This study was reviewed and deemed exempt by the institutional review board. At our institution, end-of-shift evaluations contain the competencies from the ACGME milestone-based rating scale and space for free-text comments. Evaluations can either be requested by the resident or self-initiated by the faculty member.

This study occurred over a two-year period form October 1, 2019–September 30, 2021, coinciding with the fiscal year (FY) calendars for 2020 (FY20) and 2021 (FY2021). During the first year of the study (FY20), faculty incentive compensation was not connected to resident evaluations. At the midpoint of the study period an incentive compensation plan was introduced linking the completion of end-of-shift evaluations to the year-end bonus for FY21. Faculty bonuses, which are awarded annually, are a percentage of base salary broken down by clinical and academic metrics. Points are earned to achieve the academic bonus; completion of 40 end-of-shift evaluations earns 25% of the points needed for the full academic bonus. For a faculty member at the assistant professor level receiving the full academic bonus, meeting this fulfilment translates to approximately $4,875.

We reviewed the number of end-of-shift evaluations completed in the pre-implementation to the post-implementation period and compared by the overall number of completed evaluations and the number of completed evaluations per shift by each attending physician. We determined the overall number of completed evaluations, as well as the number of completed evaluations per shift per attending and compared the number of end-of-shift evaluations completed in the pre-implementation period to the post-implementation period.

All faculty who worked at the primary teaching site and were eligible for the annual incentive compensation bonus met study criteria. All resident-supervision shifts were included in the study. Exclusion criteria included faculty not employed during the duration of the study period and faculty who did not work with residents during the study period. Only evaluations that were completed and submitted were included in this study. We performed subgroup analysis to compare for differences between male and female physicians, and junior faculty (defined for this study as within the first 10 years of an initial faculty appointment at the completion of the study period) and senior faculty. We used our institution’s residency management system, Medhub (Minneapolis, MN), which reported end-of-shift evaluations conducted by faculty of residents during the study period.

We compared the total number of evaluations completed by each faculty member during the pre- and post-implementation periods based on the Medhub data. Multiple residents may be working during a faculty shift; therefore, each faculty member may submit multiple evaluations per shift. We calculated the number of evaluations per shift based on the total number of evaluations per attending and total number of qualifying shifts. We compared each attending physician pre-implementation to post-implementation to determine any change in the total number of evaluations and the number of evaluations per shift per attending. For this initial analysis, the focus was intentionally limited to quantitative data evaluation. We analyzed data using GraphPad Prism version 9 (Graphpad Software, San Diego, CA). Pre- and post-implementation data and subgroup analysis were compared using *t*-tests with the significance level *P* < 0.05 applied.

## RESULTS

During the study period, 65 physicians submitted resident evaluations. Among them, 27 did not meet inclusion criteria for the study and were excluded. Reasons for exclusion of faculty were as follows: not employed during the entire study period (14); employed as fellows (10); or did not work at the teaching hospital for the duration of the study (3). There were 38 attendings eligible for the study; 39.5% were female and 60.5% were junior faculty. Among the junior faculty, 47.8% were female; 26.7% of the senior faculty were female. We included 2,778 resident evaluations in the study. The total number of evaluations submitted pre-implementation of the incentive metric (FY20) was 1,149. After implementation, the total number submitted (FY21) was 1,629, an increase in 42% increase for year two (*P* = 0.001).

**Figure 1a:**
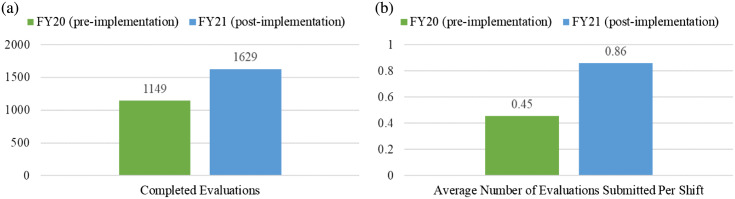
Completion of resident evaluations by faculty pre- and post-implementation of a financial-incentive metric (statistically significant, *P* = .001). **1b**. Average number of evaluations submitted per shift pre- and post-implementation of the incentive metric (statistically significant, *P* < .001).

Each individual attending shift was reviewed, and the average number of evaluations submitted per shift (for any shift worked with residents) is reported in Figure 1b. The mean number of evaluations submitted per shift pre-implementation of the incentive metric was 0.45 (SD 0.47). After implementation of the incentive metric, the mean number of evaluations submitted per shift increased to 0.86 (SD 0.56), a statistically significant increase (*P* < .001). Overall, 84.2% of faculty members had an increase in the number of evaluations submitted per shift. The breakdown by female, male, junior, and senior faculty are detailed in Table 1.

**Figure 2a:**
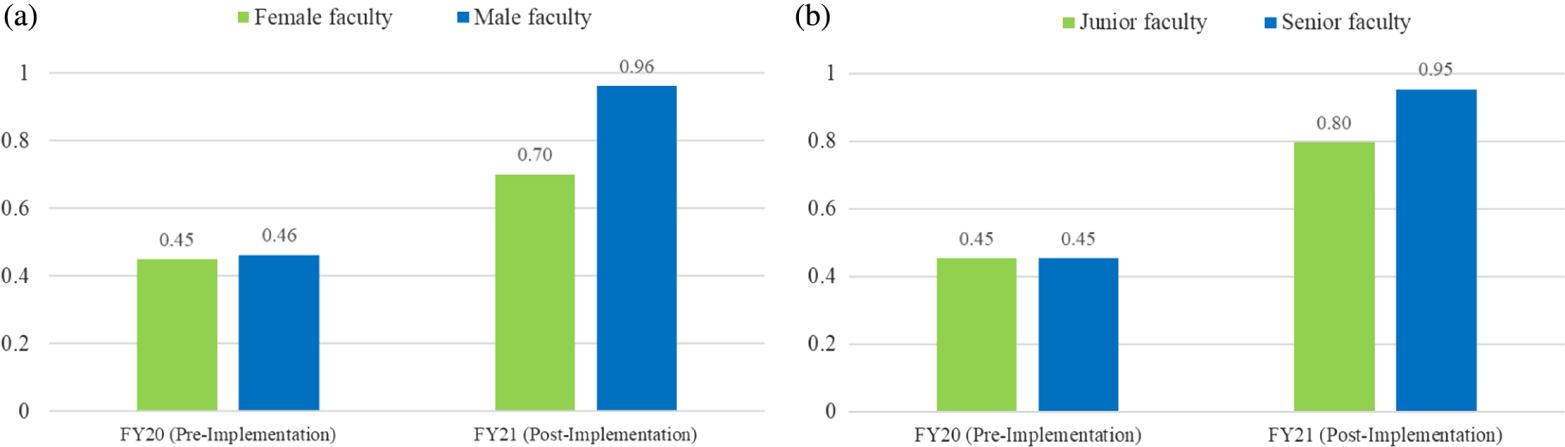
Average number of evaluations completed per shift by female and male faculty pre- and post-implementation of the incentive metric. **2b**. Average number of evaluations completed per shift by junior and senior faculty pre- and post-implementation of the financial-incentive metric.

Across all attendings who had an increased number of evaluations during the post-implementation period, the average increase was 0.5 evaluations per shift (range 0.01–1.54). Among the four who submitted fewer evaluations per shift during the intervention period, the average decrease was 0.19 evaluations per shift (range 0.01–0.55). Figure 2 compares the average number of evaluations per shift for females vs males in the pre- and post-implementation period and junior vs senior faculty. Both female faculty and male faculty had a significant increase in the number of evaluations pre- and post-implementation (*P* = .027, *P* < .0001, respectively) (Figure 2a). However, male faculty had a greater overall increase in the average number of evaluations per shift (*P* = .049). Figure 2b shows the increase pre- and post-implementation for junior and senior faculty, which was significant within both groups (*P* < 1, *P* < .001, respectively). Senior faculty completion rate had a slightly higher increase than for junior faculty; however, this did not reach statistical significance.

## DISCUSSION

In this study we identify a strategy for improving the collection of resident evaluations from faculty members at a primary academic EM residency teaching site. Specifically, the study demonstrates that the majority of attending physicians exhibited an increase in the number of evaluations they submitted per shift, resulting in a significant increase in the total number submitted by the entire study group after implementation of a faculty incentivization strategy. This incentivization strategy assisted our program to meet the ACGME requirements for frequent resident feedback. Comparison of subgroups shows that while both female and male faculty had a significant increase in the number of evaluations post-implementation, the average increase in evaluations per shift for male faculty was greater than in female faculty. This may reflect the trend of female faculty being less able to be academically productive during the COVID-19 pandemic.^11^–^13^

A variety of ways to increase faculty contributions to the myriad of instructional needs in the GME realm have been investigated. Such endeavors include assigning “value units” to scholastic contributions, sometimes termed “educational value units” (EVU) or “academic relative value units” (aRVU) to be used in a diverse manner based on departmental needs. One academic EM group concerned with the marginalization of educational pursuits developed a “mission-based budget” in which an EVU system assigns activities to faculty with aligned funding, which led to a significant improvement in the completion of obligations.^14^ Ma et al has shown that academic productivity of a faculty could be measured and, thus, fiscally rewarded based on the accruing of “academic points” vis a vis aRVUs and using an associated bonus system.^15^

A Faculty Incentive Task Force within another group of emergency physicians created educational activity categories, assigned standardized time values correlating to EVUs for each activity, and then set a threshold of total EVUs to be met in order to receive compensation, which resulted in increased faculty completion of resident and fellow monthly evaluations as well as attendance at didactic conferences.^16^ Similarly, Pugh et al demonstrated that the implementation of quarterly bonuses increased faculty participation in conferences and resident evaluations.^7^

Evaluation of residents must be well documented and trended to allow for proper summative assessment, teaching, and identification of possible interventions. Considering this, program leadership needs to curate frequent feedback to create a sufficient volume from diverse faculty across time. Thus, the focus of this investigation was intentionally limited to the quantitative analysis regarding numbers of evaluations. This was done from the perspective of program administration and the ACGME requirement for frequency/amount of feedback using the milestone scales.

## LIMITATIONS

Limitations of this study include the fact that it was conducted at a single site and only for the duration of a single fiscal year. In this study we compared the number of evaluations, not their overall quality. It is important to note that the COVID-19 pandemic occurred during the study period, causing disruptions to physician staffing and salaries nationally. Within our institution, emergency physician work hours and salaries were not reduced due to the pandemic and did not affect the faculty’s ability to submit evaluations.

## CONCLUSION

Incentivizing faculty through use of a bonus compensation structure increased the number of evaluations of residents submitted at our institution. This information may be applied by other programs to increase the number and frequency of resident evaluations.

## Figures and Tables

**Table 1:** Percentage change in number of evaluations submitted per shift after implementation of financial incentive.

	Increase	Decrease	No change
All faculty (N = 38)	84.2%	10.5%	5.3%
Female faculty (n = 15)	73.3%	13.3%	13.3%
Male faculty (n = 23)	91.3%	8.7%	0%
Junior faculty (n = 23)	82.6%	8.7%	8.7%
Senior faculty (n = 15)	86.6%	13.3%	0%
